# Optic Neuritis

**Published:** 1890-09-27

**Authors:** 


### OPTIC NEURITIS.

In the year 1840 was published an elaborate illustrated work on diseases of the eye by Mr. Frederick Tyrrell, senior surgeon to the Royal London Ophthalmic Hospital. In this work, although there is a description of retinitis, the term *neuritis* does not occur. The term neuritis, as applied to maladies of the eye, is, therefore, a comparatively modern adoption. The definition usually given of neuritis is "inflammation of a nerve generally the result of rheumatic inflammation." But Mr. Macnamara, surgeon to the Royal Westminster Ophthalmic Hospital, has recently published a paper (*British Medical Journal*) headed "Malarial Neuritis and Neuro-retinitis." Several cases are related. A gentleman who had suffered frequently from intermittent fever in India, after one attack found himself unable to read or write, but that impairment of vision gradually cleared away. The patient on return to England got wet through, experienced a paroxysm of ague, followed by such impairment of vision that he was unable to do more than dimly count his fingers. There was no pain, but the pupils acted imperfectly. "The optic discs were completely obscured by effusion, which extended into the retina." There was no evidence of either syphilis or rheumatism. Quinine was given, and subsequently arsenic and strychnine, vision gradually becoming normal. The second case reported is of very similar character. Mr. Macnamara remarks that after carefully watching cases of this kind, he inclines to the idea that the inflamed state of the optic papilla is due to something of the nature of a microbe which becomes planted in the affected tissues. Mr. Macnamara is not the only author who has referred affections of the eye to malaria. Dr. Kipp in the "American Ophthalmological Society's reports," in recently published observations on "Malarial Keratitis," in which he refers ulcerations to malaria. He describes a narrow serpiginous ulcer which extends superficially from without inwards, thrusting out branches in its progress, like a fungoid growth. Now when a person is in a reduced state of health from so-called malarial fever, various sequelæ are apt to present. But do not similar sequelæ occur after other fevers? Also in connection with scurvy and syphilis? So many maladies have been attributed to malaria, that we always feel somewhat sceptical when called upon to increase the number.

But in a second paper in the *British Medical Journal* of May 3rd, Mr. Macnamara has observations on "Rheumatic neuritis and neuro-retinitis." Cases are detailed, and in the majority of such cases the patient's history points to inherited rheumatism, and he has himself suffered from the disease. Exposure to damp and cold is generally given as the direct cause. The appearance of the inflamed papilla differs in no respect from that produced from malaria. But it would seem there is more pain, for Mr. Macnamara observes, "The pain in these cases is stated by the patient to be deep seated in the orbit, and to extend thence over the forehead and right side of the head"; "it is worse at night and increased on pressure applied over the globe of the eye." Returning to Tyrrell's work on diseases of the eye, we do not find that in his day malaria was regarded as a factor. Neither is much said of rheumatism, but the "gouty diathesis" finds a place in Tyrrell's scheme of causation. If Mr. Macnamara's views are correct, one fact stands prominently forward, viz., that a similar malady may be excited by different causes; a matter which is sometimes lost sight of. As regards optic neuritis not only has it been referred as above to malaria and rheumatism and gout, but also to lead poisoning. In a recent number of the *Lancet*, Mr. Jeaffreson, senior surgeon to the North Durham Infirmary, describes a special form of optic neuritis in association with lead poisoning. It has also been observed in connection with menstrual suppression. Sexual excesses, and alcoholic indulgence, may induce it. And it is well-known that intercranial tumours may be a cause. Exposure to the electric light is often a cause.

